# Presepsin and fetuin-A dyad for the diagnosis of proven sepsis in preterm neonates

**DOI:** 10.1186/s12879-019-4316-5

**Published:** 2019-08-06

**Authors:** Halil Değirmencioğlu, Buse Ozer Bekmez, Turan Derme, Mehmet Yekta Öncel, Fuat Emre Canpolat, Cüneyt Tayman

**Affiliations:** 1 Department of Neonatology, Private Water Vatan Hospital, Kahramanmaraş, Turkey; 2 Department of Neonatology, Zekai Tahir Burak Women’s Health Education and Research Hospital, Ankara, Turkey; 3 Faculty of Medicine, Department of Pediatrics, Division of Neonatology, İzmir Katip Çelebi University, İzmir, Turkey

**Keywords:** Presepsin, Neonatal sepsis, Diagnosis

## Abstract

**Background:**

Diagnosis is the most strenuous step in the evaluation of neonatal sepsis. No gold standard diagnostic method is available except for blood culture. We aimed to investigate the role of positive and negative acute phase reactants, namely presepsin and fetuin-A, in the diagnosis of culture-proven late-onset sepsis.

**Methods:**

A prospective, case-control study with the infants ≤32 weeks of age with a diagnosis of culture-proven late-onset sepsis was designed. Twenty-nine preterm infants with similar gestational and postnatal ages without sepsis constituted the control group. Serum values of presepsin, fetuin-A, C-reactive protein and interleukin-6 were evaluated at the enrollment, third and seventh days of the diagnosis in the infants with positive blood culture results.

**Results:**

First-day presepsin values were significantly higher in the culture-positive infants than the control group [1583 ng/L (1023–1731) vs. 426 ng/L (287–589), p = < 0.0001]. Presepsin was found to have an 88.9% sensitivity and 88.9% specificity with a cut-off value of 823 ng/ml for culture-proven LOS in our study, and area under the receiver-operating curve was 0.939. Fetuin-A levels were similar between the study and control groups (*p* > 0.05).

**Conclusion:**

Presepsin may be an accurate marker for both diagnosis and monitoring of treatment response for culture-proven late-onset sepsis in preterm infants. However, fetuin-A does not seem to be a useful tool for the diagnosis of sepsis.

## Background

Despite recent advances in neonatal medicine in the last years, sepsis is still a leading cause of mortality and morbidity among hospitalized infants in neonatal intensive care units (NICU) [1–3]. Nearly two-thirds of extremely low birth weight (ELBW) infants experience more than one episode of suspected or culture-proven late-onset sepsis (LOS) during hospitalization [1]. Neurodevelopmental impairment and mortality are substantially higher in ELBW infants with sepsis, even if without meningitis, compared with those not experienced infections [1]. Related to these high risks, if there is any clinical suspicion arised, empirical antibiotic treatment should be immediately.

Diagnosis is the most strenuous step in the assessment of sepsis beacuse of non-specific clinical signs and symptoms. Blood culture positivity is still the gold standard diagnostic tool. Blood culture technique difficulties, such as long waiting time, contamination and inability to produce microorganisms have led to a comprehensive search for biomarkers in addition to frequently used acute phase reactants like C-reactive protein (CRP) and procalcitonin for diagnosis of neonatal LOS [2–5]. Some inflammatory markers and cytokines have been investigated for the determination of an ongoing infection. However, the incidence at which each marker begins to show increasing and the time to normalization differs through each marker [6–8].

Cluster of differentiation (CD) 14 is the co-receptor of lipopolysaccharide-lipopolysaccharide binding protein (LPS-LBP) complexes, together with Toll-Like Receptor (TLR) 4 and MD-2. With stimulation by pathogen microorganisms, a soluble CD14 subtype called presepsin is released into the circulation from the surface of membranes of immune cells and also actively secreted from these tissues [9, 10]. This molecule transduces the endotoxin signal, thus leading to the release of cytokines such as tumor necrosis factor-α, interferon-γ, interleukin-1β, interleukin-8, and interleukin-6(IL-6), causing systemic inflammatory response syndrome [9]. Although the function of presepsin is not precisely clear, it possibly regulates the immune response by interaction with lymphocytes. High levels of presepsin in blood circulation are thought to reflect systemic inflammation and therefore may be a reliable diagnostic marker in sepsis and systemic inflammatory response. Moderate diagnostic accuracy was demonstrated in the presence of sepsis by a meta-analysis in adult population [11]. Afterward, the diagnostic value in neonates was investigated in various reports [12–17]. Recently Bellos et al. demonstrated that presepsin was a sensitive biomarker in neonatal sepsis [18].

Fetuin-A (also termed alpha-2-HS-glycoprotein) functions as a negative acute-phase protein that is down-regulated in case of inflammation [19]. Most of the data about its function was obtained from animal studies. It was shown that fetuin-A levels decline in the early period of sepsis, but begin to increase again at 72 h of inflammation [20, 21]. It was demonstrated that fetuin-A supports the functioning of the immune system partially by inhibiting the release of late mediators of inflammation. Although it is still unclear how fetuin-A induces suppression of late mediators, it is believed to play a crucial role in the struggle against sepsis. There is limited data on whether fetuin-A is helpful in the diagnosis and follow-up of neonatal sepsis.

We aimed to evaluate the diagnostic value of presepsin and fetuin-A dyad as a diagnostic tool for LOS in preterm infants.

## Methods

### Patient population

This study was performed in the NICU between January and March 2018. Our unit is a huge perinatal center that has approximately 18,000 annual births, 4000 patient admissions, and 100 tertiary level NICU incubators. The local ethics committee confirmed this study. It was carried out according to the principles of the Declaration of Helsinki and was approved by the parents of each patient.

### Study design

Infants who were born ≤32 weeks gestational age and 4 to 60 days postnatal age with gram-positive and/or negative bacteria detected in blood culture were included in the study. Exclusion criteria were the presence of major congenital and/or chromosomal anomalies. Patients were matched in terms of gestational age, birth weight and gender. Newborn infants who had no signs or symptoms of sepsis were enrolled in the control group.

Laboratory tests included complete blood count, CRP, IL-6, Presepsin, Fetuin-A, blood, urine and cerebrospinal fluid (CSF) cultures. These tests were performed on the first evaluation day before antibiotic treatment and repeated after 48 and 120 h from the first sampling. Blood samples for Presepsin and Fetuin-A were stored at − 80 °C until blood culture results were gathered. Blood cultures were not taken from control group patients.

### Infection definitions

Diagnosis of sepsis was put forward based on the report of the expert meeting on neonatal and pediatric sepsis [22]. Clinical signs were often subtle and insidious and include respiratory (tachypnea, apnea, increase in ventilatory support), metabolic (feeding problems, temperature irregularity, metabolic acidosis), circulatory (hypotension, poor perfusion and decrease in heart rate) and neurologic (lethargy, irritability) symptoms. The presence of at least two clinical findings and at least two laboratory signs with accompanying negative blood culture was defined as ‘probable sepsis’. Sepsis occurring after 72 h of life was diagnosed as late-onset sepsis. Patients were diagnosed with ‘culture-proven sepsis’ when the blood culture was positive in addition to clinical signs and abnormal acute phase reactants.

### Infection biomarkers

Plasma sample of 0.5 ml was gathered in an ethylene diamine tetra-acetic acid (EDTA) containing tube from venous puncture, and after centrifugation samples were stored at − 80 °C. Presepsin was detected in these samples simultaneously with the sepsis investigation. Presepsin was quantified by chemiluminescent enzyme immunoassay with the automated analyzer Human sCD14 enzyme-linked immunosorbent assay (ELISA) Kit (Elabscience Biotechnology Co.®, Ltd., China). Serum fetuin-A levels were confirmed by a sandwich ELISA in sepsis and control groups (Human Fetuin-A ELISA kit, Biovendor®, Modrice, Czech Republic).

Tina-quant CRP (latex) high sensitive immunoturbidimetric assay on the Roche Modular P analyzer was used to measure plasma CRP concentrations according to the manufacturer’s instructions (CRP latex HS, Roche kit, Roche Diagnostics®, Mannheim, Germany). CRP was considered normal if it was in the 0–5 mg/L range. Serum levels of IL-6 were analyzed by IL-6 solid phase, enzyme labeled, chemiluminescent sequential immunometric assay on an IMMULITE 1000 analyzer, according to the manufacturer’s instructions (Siemens Diagnostic Product Corporation®, Los Angeles, CA). IL-6 levels > 25 pg/ml were regarded as abnormal. The fully automated BACTEC method by BacT/Alert (Biomeriux®, France) was used for cultures.

Empirical penicillin G and gentamicin therapy are given to high-risk preterm newborns; including preterm premature rupture of the membranes, maternal chorioamnionitis, fetal/intrapartum stres, surfactant requirement admitted to our clinic. Initial antibiotic regimens included intravenous vancomycin and amikacin when there was suspicion of LOS. Antibiotic therapy protocol was assigned according to the most common infectious agents in our NICU and switched relying on isolated microorganisms in blood culture and antimicrobial susceptibility test results. The most common pathogens of LOS in preterm neonates were coagulase-negative staphylococci, Klebsiella pneumonia and Serratia marcescens. While the duration of therapy was 10 days in case of culture-proven LOS, 14 days of treatment was assured if there was a specific focus of infection such as meningitis.

### Statistical analysis

SPSS for Windows® (version 20.0) statistical package was used for statistical analyses. The χ2 test was utilized for comparison of categorical variables between groups. The normal distribution assumption of numerical variables was evaluated with the Kolmogorov–Smirnov test.

T-test was used for normally distributed variables and Mann Whitney U test for abnormal distributed or non-parametric variables. ANOVA and Kruskal-Wallis test were used for the evaluation of the difference in normally and abnormal distributed variables respectively if there were three or more variables. ROC curve was used for determination of the diagnostic ability of different variables. Best cut-off values were calculated with the Youden index. A standard *P* value of < 0.05 indicated statistical significance.

## Results

A total of 55 patients were included in the study. Of these, 26 had culture-proven LOS. Twenty-nine patients constituted the control group. Mean gestational ages and birth weights were 29.1 ± 3.7 and 29.7 ± 1.8, 1202 ± 698 and 1212 ± 268 g in sepsis and control groups, respectively. Gram-negative bacteria were detected in 17 of the 26 infants with sepsis. The demographic and clinical features of the study and control groups are shown in Table 1. There were no differences between the groups in terms of maternal, demographic or clinical properties.

**Table 1:** Demographic and clinical characteristics of study and control group

	Sepsis group (*n* = 26)	Control group (*n* = 29)	*P*
Gestational age, w	29.1 ± 3.7	29.7 ± 1.8	> 0.05
Birth weight, g	1201 ± 698	1212 ± 268	> 0.05
Male gender n, (%)	12 (66)	15 (55)	> 0.05
Mode of delivery (Cesarean), (%)	14 (77.7)	17 (63)	> 0.05
PPROM (%)	1 (5.5)	2 (7.4)	> 0.05

Serum levels of presepsin, fetuin-A, CRP and IL-6 on three different days were compared. Initial CRP, IL-6 and presepsin concentrations were higher in septic neonates compared with the control group (Table 2, *p* = 0.001, *p* = 0.004 and *p* = 0.000 respectively). Fetuin-A levels were similar in sepsis and control groups (*p* = 0.423).

**Table 2:** Comparison of initial day WBC, CRP, IL-6, Presepsin and Fetuin-A measurements between study and control groups

	Sepsis group (*n* = 26)	Control group (*n* = 29)	*P*
First day			
WBC_1,_ (10^3^/μL)	12.6 ± 5.9	16.1 ± 5.3	**< 0.05**
CRP_1,_ (mg/L)	24.84 ± 31.76	2.25 ± 2.09	**< 0.01**
IL-6_1,_ (pg/ml)^a^	190 (11–5000)	13.6 (0–42)	**< 0.01**
Presepsin_1,_ (ng/L)	1583 (1023–1731)	426 (287–589)	**< 0.01**
Fetuin-A_1,_ (ng/ml)	32.66 ± 6.05	31.33 ± 8.57	0.42

^a^median (min-max)

Gram-negative bacteria were detected in 17 of 26 culture-proven LOS patients. The microorganisms were *Klebsiella pneumonia* in seven cases, *Escherichia coli* in five cases, *Acinetobacter baumannii* in three cases, *Pseudomonas aeruginosa,* and *Serratia marcescens* in one case each. *Staphylococcus epidermidis* was isolated from the blood cultures of nine infants with LOS. No difference was revealed concerning presepsin, fetuin-A, CRP, IL-6 and white blood cell (WBC) between gram negative and positive bacteria subgroups (Table 3).

**Table 3:** Comparison of serial WBC, CRP, IL-6, Presepsin and Fetuin-A measurements between gram (+) and gram (−) sepsis groups

	Gram (+) sepsis (*n* = 9)	Gram (−) sepsis (*n* = 17)	*P*
First day			
WBC_1,_ (10^3^/μL)	12.85 ± 4.93	12.45 ± 6.65	> 0.05
CRP_1,_ (mg/L)	13.95 ± 11.5	32.48 ± 28.35	> 0.05
IL-6_1,_ (pg/ml)*	173 (11–4879)	212 (49–5000)	> 0.05
Presepsin_1,_ (ng/L)	1385 (988–1427)	1471 (1088–1731)	> 0.05
Fetuin-A_1,_ (ng/ml)	35.20 ± 7.33	31.93 ± 4.39	> 0.05
Third day			
WBC_2,_ (10^3^/μL)	12.88 ± 4.29	13.62 ± 5.53	> 0.05
CRP_2,_ (mg/L)	16.51 ± 9.00	22.80 ± 16.77	> 0.05
IL6_2,_ (pg/ml)*	41.3 (11.7–99.7)	53 (8.4–103)	> 0.05
Presepsin_2,_ (ng/L)	1030 (826–1292)	1101 (931–1315)	> 0.05
Fetuin-A_2,_ (ng/ml)	32.94 ± 5.89	31.12 ± 4.39	> 0.05
Seventh day			
WBC_3,_ (10^3^/μL)	14.65 ± 9.41	10.15 ± 2.22	> 0.05
CRP_3,_ (mg/L)	5.92 ± 4.07	11.03 ± 5.36	> 0.05
IL-6_3,_ (pg/ml)^a^	12.5 (6–84)	16 (2–44)	> 0.05
Presepsin_3,_ (ng/L)	608 (238–854)	781 (452–873)	> 0.05
Fetuin-A_3,_ (ng/ml)	37.05 ± 4.69	32.50 ± 4.48	> 0.05

^a^median (min-max)

**Fig. 1:**
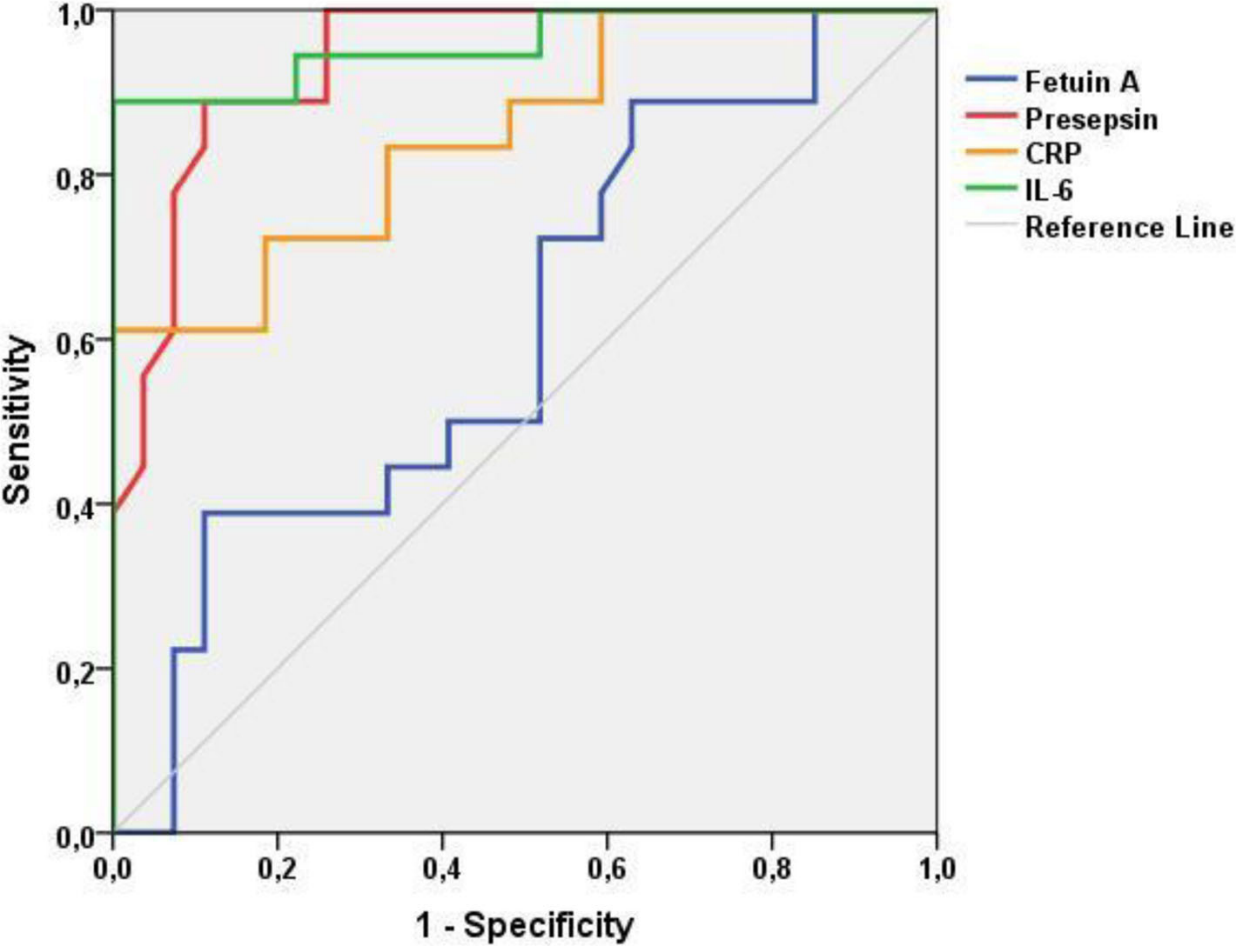
ROC curve of presepsin at enrollment in proven sepsis

Receiver operating characteristic curve (ROC) was utilized for the detection of the area under the curve (AUC) for CRP, IL-6 and presepsin. Cut-off values for both parameters were determined through ROC curve analysis. Positive and negative predictive values were both calculated. ROC curves of the two groups are shown in Fig. 1. The AUC was 0.939 for presepsin, and 0.959 and 0.850 for IL-6 and CRP, respectively (Table 4).

**Table 4:** The AUC, Cut-off, specificity and sensitivity of CRP, IL-6, Presepsin and Fetuin-A for sepsis group vs. control group

	AUC	Cut-off level	Specificity (%)	Sensitivity (%)	PPV (%)	NPV (%)
CRP_,_ mg/L	0.850	3.90	81.5	72.2	73.6	81.4
IL-6_,_ pg/ml	0.959	23.22	78.2	94.4	75	95.4
Presepsin_,_ ng/L	0.939	823	88.9	88.9	72.7	87.5
Fetuin-A_,_ ng/ml	0.612	30.17	48.1	72.2	48.1	68.4

## Discussion

We investigated the role of the presepsin and fetuin-A dyad for diagnosis and follow-up of culture-proven LOS in preterm infants. While we found significantly higher presepsin values in LOS, fetuin-A concentrations were almost similar between the groups.

Early diagnosis is often compelling due to the lack of overt and specific signs and symptoms [2, 3]. Moreover, identification is much more troublesome owing to the already sick state and accompanying morbidities. Nevertheless, high mortality rate and long-term adverse neurodevelopmental sequelae make prompt and exact diagnosis compulsive in LOS [1–3]. The only ‘gold standard’ diagnostic method is a positive blood culture in neonatal sepsis. But sample volume and bacterial load in blood influence the accuracy of the test result. The necessity of a long time for a blood culture result is another obstacle in diagnosis. Various markers have been identified so far, but an ideal marker has not yet been found [23, 24]. The dilemma associated with CRP is the poor sensitivity and delay in elevation after an infective stimulus [4]. These challenges necessitate the use of molecular-based techniques, like PCR, for diagnosis [2, 23, 24]. We planned to explore the diagnostic role of the presepsin and fetuin-A dyad in culture-proven LOS in preterm infants. Presepsin was demonstrated to be a valuable tool for early diagnosis of sepsis.

Presepsin was discovered as a hopeful diagnostic marker of sepsis for the first time in 2004 [25]. Consecutive studies concentrated on the strength and diagnostic value of presepsin compared with the most widely used sepsis markers such as CRP and procalcitonin. Two serial meta-analyses revealed that presepsin is a powerful diagnostic marker, but fails in the verification or exclusion of a diagnosis if used alone in the adult population [11, 26]. A very recent meta-analysis showed evidence of 0.91 sensitivity and 0.97 specificity of presepsin in neonatal sepsis, especially if the cut-off value is between 650 and 850 pg/ml [18].

Presepsin values were defined both in critical preterm and term neonates with/without sepsis in different studies [13, 14, 18]. Mussap et al. defined a mean value of 643.1 ng/L for presepsin in non-septic preterm neonates [27]. They also showed no link between gestational age and presepsin values and presented the ranges of presepsin in septic neonates. Later, the same researchers showed that presepsin was a useful marker for discrimination of bacterial sepsis from non-bacterial systemic inflammatory response syndrome with 100% sensitivity and 81.2% specificity with a cut-off value 548 ng/L in the presence of sepsis.

Presepsin was found to have 88.9% sensitivity and 88.9% specificity with a cut-off value of 823 ng/ml for culture-proven LOS in our study. This was similar to the literature. Poggi et al. demonstrated that presepsin was a successful marker in the early period of LOS with a cut-off value of 885 ng/l, having 100% specificity and 94% sensitivity [13]. Additionally, it was shown to be a valuable tool for follow-up of response to antibiotic therapy, even if used alone. However, ROC curves cannot be established for comparison due to the unavailability of CRP and procalcitonin in control groups.

Fetuin-A is a hepatic serum protein that inhibits pathological calcification [28]. Although the exact role is not yet thoroughly understood, fetuin-A is speculated to act as a negative acute phase reactant [19, 20]. Regulation of fetuin-A differs in the early and late periods of sepsis due to various proinflammatory cytokines. In animal studies, it was shown to decrease as a response to the early inflammatory process, reaching normal values with time, possibly due to late mediators [28]. This molecule was considered to be a hopeful marker for sepsis after proteomic studies of lipopolysaccharide-stimulated secretory proteins in endothelial cells [29]. A limited number of clinical studies supported this conclusion. Recently, Karampela et al. revealed an inverse relationship of CRP and IL-6 in adults, compatible with the findings of the previous studies [30].

Fetuin-A reaches peak serum concentrations in fetal life, possibly reflecting a crucial role in tissue formation [20]. Again, high levels of extremely preterm infants authenticated this hypothesis in a few studies. There are a limited number of studies defining the normal values of fetuin-A in neonates [31]. Häusler et al. measured serum values in healthy children of various ages, including preterm and term infants [20]. The concentrations reached peak values in premature infants with 23–30 gestational weeks (1 ± 0.33 mg/ml). Briana et al. investigated maternal, fetal and neonatal plasma levels simultaneously in term intrauterine growth retardation (IUGR) and appropriate for gestational age (AGA) infants [20]. No difference was seen between AGA and IUGR infants (43.2 + 15.7 ng/mL vs. 45.2 + 16.4 ng/mL, respectively). In a small number of studies, wide differences were present in fetuin-A plasma concentrations. Our values are similar to those detected in healthy term infants in the study of Briana et al. [30]. But there is no information on the range of fetuin-A in neonatal sepsis. Hence, we could not compare our values with that of others. We can only conclude that fetuin-A levels did not wax and wane during antibiotic treatment. Therefore, it does not appear to be a useful marker for follow-up based on our currently available data.

Our study has some limitations. First, the small sample size may have prohibited the emergence of an overt change in plasma fetuin-A concentration. Again, our study included preterm infants only with culture-proven sepsis. If the patients with clinical sepsis were incorporated into the study, we could compare values of the two groups. Future studies must focus on the diagnostic role of fetuin-A in neonatal sepsis with larger cohorts.

This study is original in terms of the analysis of two markers simultaneously. For low-resource settings with high admission and infection rates in NICU, presepsin may be promising with higher sensitivity and similar cost with interleukin-6, which is routinely used with the aim of sepsis work-up in our clinic. This study demonstrated that presepsin is a useful diagnostic marker of preterm LOS in comparison to CRP and IL-6. It may also be helpful in monitoring the antibiotic response. But no difference was seen in serum fetuin-A concentrations between groups.

## Conclusion

This is the first study investigating the role of the dyad markers presepsin and fetuin-A simultaneously in the diagnosis and monitoring of the success of therapy in culture-proven LOS in preterm infants. Presepsin seems to be a useful marker for late-onset sepsis in preterm infants based on the results of our study. However, fetuin-A levels do not correspond with already used acute phase reactants or presepsin.

## Data Availability

Please contact the corresponding author for data requests.

## Abbreviations

AGA: Appropriate for gestational age
AUC: Area under the curve
CRP: C-reactive protein
CSF: Cerebrospinal fluid
EDTA: Ethylene diamine tetra-acetic acid
ELBW: Extremely low birth weight
ELISA: Enzyme-linked immunosorbent assay
IL-6: Interleukin-6
IUGR: Intrauterine growth retardation
LOS: Late-onset sepsis
NICU: Neonatal intensive care unit
ROC: Receiver operating characteristic curve
SPSS: Statistical package for the social sciences
WBC: White blood cell
